# Rhizosphere microbial community assembly and association networks strongly differ based on vegetation type at a local environment scale

**DOI:** 10.3389/fmicb.2023.1129471

**Published:** 2023-03-14

**Authors:** Luxian Liu, Liya Ma, Mengmeng Zhu, Bo Liu, Xu Liu, Yu Shi

**Affiliations:** ^1^State Key Laboratory of Crop Stress Adaptation and Improvement, School of Life Sciences, Henan University, Kaifeng, Henan, China; ^2^State Key Laboratory of Soil and Sustainable Agriculture, Institute of Soil Science, Chinese Academy of Sciences, Nanjing, China

**Keywords:** vegetation type, rhizosphere microbes, microbial networks, local scale, bacteria and fungi

## Abstract

**Introduction:**

Rhizosphere microbes perform critical functions for their hosts, and their structure is strongly influenced by vegetation type. Although studies on the effects of vegetation on rhizosphere microbial community structure have been conducted at large and global environment scales, studies at local environment scales would eliminate numerous external factors such as climate and soil type, while highlighting the potential influence of local vegetation type.

**Methods:**

Here, we compared rhizosphere microbial communities using 54 samples under three vegetation types (herb, shrubs, and arbors, with bulk soil as the control) at the campus of Henan University. 16S rRNA and ITS amplicons were sequenced using Illumina high throughput sequencing.

**Results and Discussion:**

Rhizosphere bacterial and fungal community structures were influenced considerably by vegetation type. Bacterial alpha diversity under herbs was significantly different from that under arbors and shrubs. The abundance of phyla such as Actinobacteria was extremely higher in bulk soil than in the rhizosphere soils. Herb rhizosphere harbored more unique species than other vegetation type soils. Furthermore, bacterial community assembly in bulk soil was more dominated by deterministic process, whereas the rhizosphere bacterial community assembly was dominated by stochasticity and the construction of fungal communities was all dominated by deterministic processes. In addition, rhizosphere microbial networks were less complex than bulk soil networks, and their keystone species differed based on vegetation type. Notably, bacterial community dissimilarities were strongly correlated with plant phylogenetic distance. Exploring rhizosphere microbial community patterns under different vegetation types could enhance our understanding of the role of rhizosphere microbes in ecosystem function and service provision, as well as basic information that could facilitate plant and microbial diversity conservation at the local environment scale.

## Highlights

– Rhizosphere microbial community structure influenced significantly by vegetation type.
– Contrasting bacterial and fungal assembly processes in bulk and rhizosphere soil.
– Rhizosphere communities harbor less complex networks than bulk soil.
– Rhizosphere bacterial communities significantly correlated with plant phylogeny.

## 1. Introduction

The rhizosphere is a hotspot of interactions between plant root and soil (Lundberg et al., 2012; Korenblum et al., 2020) It is a complex ecosystem that can be influenced considerably by the composition of the aboveground plants (Liu et al., 2020). Recently, the influence of plants on rhizosphere microbes has been studied extensively (Schmid et al., 2019; Escudero-Martinez et al., 2022) across different ecosystems, including forest (Chen et al., 2018), grassland (Birgander et al., 2017), and cropland (Simonin et al., 2020). The rhizosphere ecosystem is highly complex, and under the influence of various plant species. Therefore, the influence of vegetation type on the rhizosphere microbial community structure should be taken into account.

Recently, researchers have begun focusing on the effect of vegetation type on rhizosphere. In addition, some researchers have explored the structure and function of the global rhizosphere microbiome (Davison et al., 2015; Xu et al., 2018; Ling et al., 2022); however, the researchers mainly focused on certain plant types, or plants in multiple ecosystems. Therefore, the effects of associated factors, such as climate and soil type, on the rhizosphere microbiome cannot be eliminated at large scales.

Rhizosphere microbes, which mainly include bacteria and fungi, are essential for plant growth and development (Huang et al., 2014). Some of the microbes enhance the capacity of plants to obtain nutrients from soil, and resistance to various biotic and abiotic stress factors, such as disease (Song et al., 2021), high salinity (Schmitz et al., 2022), and drought (de Vries et al., 2020) and adaptation to changing environments (Berendsen et al., 2012; Trivedi et al., 2020). In return, the microbes get certain benefits from plants, including nutrients such as carbon (Bais et al., 2006). Consequently, vegetation type can influence rhizosphere community diversity and composition. For example, arbuscular mycorrhizal fungal colonization is higher in forbs than in grass (Bunn et al., 2015). Furthermore, in natural mountain forests of eastern China, Yang et al. (2019) observed dissimilarities in rhizosphere microbial community structure under different vegetation types, which increased significantly with an increase in plant phylogenetic distance, highlighting the role of plant phylogeny in rhizosphere community structure.

In addition to exhibiting high diversity, rhizosphere microbes establish complex ecological networks, which can also be affected by vegetation type. For instance, the rhizosphere bacterial network structure in rubber forest soils is simpler than that in tropical rainforests, whereas the rhizosphere fungal network structure in rubber forest soils is more complex (Lan et al., 2022). However, microbial community structure is the product of interactions among multiple factors, including plant factors and environmental factors (Jiang et al., 2020; Yang et al., 2021b). Consequently, large-scale studies involve too many abiotic and abiotic factors. Generally speaking, plant species is one of the key factors affecting the rhizosphere bacterial species, that is, different plant species should have different rhizosphere bacterial communities (Fitzpatrick et al., 2018). However, recent common garden experiments had found that species identity could only explain a small part of the difference in rhizosphere bacterial community (Leff et al., 2018). And the differences in rhizosphere bacterial communities of the same plant species growing in different soils are generally greater than those observed between different plant species growing in the same soil (Vieira et al., 2020). Guajardo-Leiva et al. (2022) found that soil is the main source of microorganisms, which leads to the homogeneity of community composition of different plant species growing at the same sampling point. While it remains unclear whether the same plants can exert the effects on rhizosphere community structure in different localities with highly similar climate and soil conditions frequently affected by human activities. Besides, considering community complexity would affect community dynamics, in the present study, we have adopted a metric called “Cohesion” for quantifying the degree of connectivity in microbial communities (Herren and McMahon, 2017).

Understanding the microbial assembly process is a key issue in microbial ecology, and can enhance our understanding of the mechanisms of regulation of microbial community structure (Stegen et al., 2013a; Dini-Andreote et al., 2015). Microbial community assembly occurs *via* two key processes, including stochastic processes, which mainly includes dispersal limitation, shift, and other random community changes (Hubbell and Borda-De-Agua, 2004), and deterministic processes, which is largely selection by environmental factors (Stegen et al., 2012, 2013b, 2015). Recently, numerous studies have quantified the relative importance of the two processes in community structuring (Aad et al., 2014; Feng et al., 2018b; Yang et al., 2021a; Liu et al., 2023). For example, Fan et al. (2018) investigated microbial community assembly processes in the rhizosphere soils of wheat fields. Furthermore, some researchers have explored the microbial community assembly processes in the vadose zone (Sheng et al., 2021).

The two dominant processes, stochasticity and determinism, have been further disentangled into five plausible scenarios (Shi et al., 2020a), including heterogeneous selection (HeS) and homogeneous selection (HoS), which belong to determinism (Dini-Andreote et al., 2015), and homogeneous dispersal (HD; van der Plas et al., 2018), dispersal limitation (DL; Whitaker et al., 2003; Zhou et al., 2008), and undominated (UD) cases, which belong to stochastic processes (Jiao et al., 2020). Exploration of microbial community assembly based on the five key processes above could enhance the understanding of rhizosphere microbial community structure across different vegetation types.

City parks or university campuses, which exhibit high plant species diversity, are ideal platforms for investigating the influence of vegetation type on rhizosphere microbes at relatively small spatial scales. In the present study, we collected 54 samples from two locations at Henan University to analyze rhizosphere microbial community structure across three vegetation types.

We hypothesized that microbial community structure in bulk and rhizosphere soils is controlled by different assembly process, and the responses of bacteria and fungi vary based on vegetation type and phylogeny, with markedly different community assembly patterns. We constructed the microbial community networks under different vegetation types to determine whether their topological characteristics and core species, and the underlying factors. The results of the present study could provide novel insights on rhizosphere microbial community structure under different vegetation types at the local environment scale.

## 2. Materials and methods

### 2.1. Study site

This study was conducted using soil obtained from the park of Jinming campus of Henan University (longitude: 114.35°E, latitude: 34.80°N) in Kaifeng, China. Kaifeng has a temperate monsoon climate, with an annual average temperature of 14°C and an annual average precipitation of 650 mm. Rainfall mainly occurs in July and August every summer. The soil types mainly include fluvo-aquic soils, saline soil, aeolian sandy soil, and alluvial soils.

### 2.2. Soil sampling and testing

We selected two gardens to collect soil samples. One location is in the south of the campus and another is in the north (Supplementary Figure S1). In each garden, we collected samples of 9 species of plants. Only four species (*Ligustrum lucidum, Forsythia viridissima, Oxalis corniculate, Veronica persica*) were collected in both sampling locations. Finally, they were grouped into four vegetation types (arbors, shrubs, herbs, and bulk soil. For the detail information, please see Supplementary Table S1). No specific permissions were required for sample collection and the filed study did not involve endangered or protected species. The distance between two sampling points was more than 3 m. At first, the top litter layer was removed. Before sampling, the sampling tools were wiped with the original soil in the area near the collection location to minimize external interference as much as possible. Subsequently, while wearing disposable gloves, the soil was gently dug with a shovel and the fresh soil sorted to remove stones and to find fine roots (diameter ≤ 2 mm). Taking ginkgo as an example, 0–1 m away from the trunk is the area where fine roots are predominantly distributed, and the range for arbors and shrubs is within 5–20 cm underground. When herbaceous plant samples were collected, the whole plant was taken out as completely as possible to look for fine roots. After shaking off the loose soil, the soil adhering to the fine roots over a 1-mm layer was brushed off and collected as the rhizosphere soil sample. All the soil samples were put in sterile bags (stored in dry ice boxes), transported back to the laboratory within 2 h, and then stored at −80°C until DNA extraction. Bulk soil with no plant roots collected in the adjacent area, at a distance more than 5 m from the nearest sampling point was collected as the control soil sample.

### 2.3. DNA extraction and PCR amplification

A Power Soil DNA kit (MO BIO, Carlsbad, CA, United States) was used to extract the total DNA from soil samples. Afterward, a NanoDrop ND-1000 spectrophotometer (Thermo Scientific, Waltham, MA, United States) was used to quantify the DNA concentrations of samples. The extracted DNA was diluted to approximately 25 ng/μL with distilled water and stored at −20°C until use.

Rhizosphere and bulk soil bacterial and fungal community were tested by high-throughput sequencing techniques at IlluminaNovaSeq platform of MAGIGENE Company, Guangdong, China.^1^ The V3–V4 hypervariable regions of bacterial 16S rRNA genes were amplified with the 338F and 806R primer set (Xu et al., 2016). And the ITS2 region of fungi was amplified using the ITS3F and ITS4R primer set (Toju et al., 2012; Supplementary Table S2). The Polymerase Chain Reactions (PCR) were implemented as follows: 3 min of denaturation at 95°C, followed by 30 cycles of 30 s at 95°C, 30 s at 55°C for annealing, and 45 s at 72°C for elongation, with a final extension at 72°C for 10 min. The reactions were carried out in 20-μL triplicate mixtures, each containing 4 μL of 5× FastPfu Buffer, 2 μL of 2.5 mM dNTPs, 0.8 μL of each primer (5 μM), 0.4 μL of FastPfu Polymerase, and 10 ng of template DNA (Toju et al., 2012; Xu et al., 2016).

### 2.4. Statistical analysis

The raw data sequences were processed and analyzed using QIIME2 (Bolyen et al., 2019) based on the workflow at https://qiime2.org. Briefly, to obtain the Amplicon Sequence Variant (ASV) table, quality control of the raw sequencing data was performed using the Deblur tool (Amir et al., 2017) and clustered based on 100% shared identity. The taxonomy of each bacterial phylotype was identified using the Greengenes release database (DeSantis et al., 2006) and the fungal taxonomy assignment was performed using the Sklearn-based taxonomy classifier with the dynamic Unite database from 10 October 2017.^2^ Finally, we obtained 12,730,609 bacterial sequences in and 9,153,794 fungal sequences, with 98.5% classified into 104,824 distinct ASVs in bacteria and 13,233 in fungal distinct ASVs. To rarify all datasets for each sample to the same degree, 1,122,000 and 3,089,100 bacterial and fungal sequences, respectively, were selected randomly.

### 2.5. Rhizosphere soil fungal and bacterial community structure analyses

In order to measure the difference between groups, the relative abundance of the top 10 microorganisms were logarithm transformed and then we used the LSD method of “agricolae” package for post-hoc test. To assess the abundance and diversity of microbial communities, the Shannon, Simpson, Chao1, and Observed species indices were calculated at the ASV level using QIIME2. The vegan package was used to calculate the β-diversity (Bray-Curtis and Jaccard distance) of bacterial and fungal communities in the arbor, shrub, and herbage rhizosphere soils, and bulk soil (Oksanen et al., 2020). Differences in diversity among the samples were analyzed using the Wilcoxon rank-sum test. Nonmetric multidimensional scaling (NMDS) ordinations was generated to distinguish the distribution of the samples based on Jensen-Shannon divergence (JSD), using the vegan package (Oksanen et al., 2020). Differences between communities were evaluated using Permutational Multivariate Analysis of Variance using the “Adonis” function in the vegan package.

### 2.6. Phylogenetic network construction and distance estimation

To construct the phylogenetic networks of the 14 plant species, plastome sequences were downloaded from the GenBank database (Supplementary Table S3). Maximum Likelihood (ML) analysis was performed using CIPRES Science Gateway v3.3 (Miller et al., 2010) and RAxML v8.1.11(Stamatakis et al., 2008), with GTR + T + G as the optimal substitution model. The default parameter settings were used, except for the bootstrap iterations being set to 1,000. The phylogenetic distance between each species was calculated in the PAML program (Yang, 2007).

### 2.7. Analyzing the rhizosphere soil bacteria assembly processes

According to Stegen et al. (2013b, 2015), the ß-NTI and Bray-Curtis-based Raup-Crick metrics (RC-Bray) methods were jointly used assess the community assembly processes. β-NTI measures the deviation of the β-mean nearest taxon distance (β-MNTD) and the β-MNTD of the null model, and both were calculated using Phylocom v42 (Webb et al., 2008).

Traits regulating community assembly processes should be phylogenetically conserved (Stegen et al., 2012). Therefore, a phylogenetic signal analysis is required before calculating β-NTI. The relationship between phylogenetic distances of pairwise ASVs and the corresponding environmental conditions was evaluated using “mantelcorrelog” (Stegen et al., 2012), based on the phylogenetic distances calculated using the “cophenetic” function in the “picante” package in R v4.1.3 (R Foundation for Statistical Computing, Vienna, Austria). The Euclidean distance of each soil variable of pairwise ASVs was calculated and the abundance-weighted mean value obtained. Significant relationships within a short phylogenetic distance indicate that phylogenetic signals are also significant. | β-NTI | > 2 indicates a community that is dominated by deterministic processes (Stegen et al., 2012). Conversely, | β- NTI | < 2 indicates that stochastic processes, including DL, HD, and UD, are dominant in the community (Stegen et al., 2015; Tripathi et al., 2018; Feng et al., 2018b).

In the present study, the bacterial ASVs with relatively high abundances (i.e., > 0001%) were selected (3,000 ASVs in our study) for use in calculating the β-NTI and RCbray values (Shi et al., 2018; Feng et al., 2018a).

### 2.8. Analyzing rhizosphere soil fungal and bacterial stability

Co-occurrence network analyses were conducted based on a SparCC correlation matrix using the SpiecEasi package in R (Friedman and Alm, 2012; Kurtz et al., 2015). To enhance the reliability of the networks, the ASV table was filtered. We constructed four networks corresponding to the four types of samples, including the rhizosphere arbor, shrub, and herb soil, and bulk soil. We only retained ASVs present in more than 20% of the bacterial samples and more than 30% of the fungal samples for each sample type. For each group, the selected fungal and bacterial ASVs were used to jointly construct the microbial network, with 533, 401, 393, and 754 ASVs retained in the herb, shrub, and arbor rhizosphere soil, and bulk soil, respectively, for network construction.

According to Banerjee et al. (2018), network hubs, module hubs, and connectors were defined as keystone species in the present study (Shi et al., 2020b). In addition, according to Guimerà and Nunes Amaral (2005), the z-scores (within-module degree) and c-scores (participation coefficient) of each node in the networks were calculated to identify the hubs and connectors. Based on the threshold values of the within-module degree (z-score) and participation coefficients (c-score) of nodes, nodes with a z-score > 2.5 and c-score > 0.6 were defined as network hubs. Nodes with a z-score > 2.5 and c-score < 0.6 were defined as module hubs, whereas nodes with a z-score < 2.5 and c-score > 0.6 were considered as connectors. Furthermore, nodes with a z-score < 2.5 and c-score < 0.6 were classified as peripherals. Shi et al. (2020b) has expounded in detail the particular role of each type of node in community networks.

## 3. Results

### 3.1. Effect of vegetation type on soil microbial community composition

**Figure 1:**
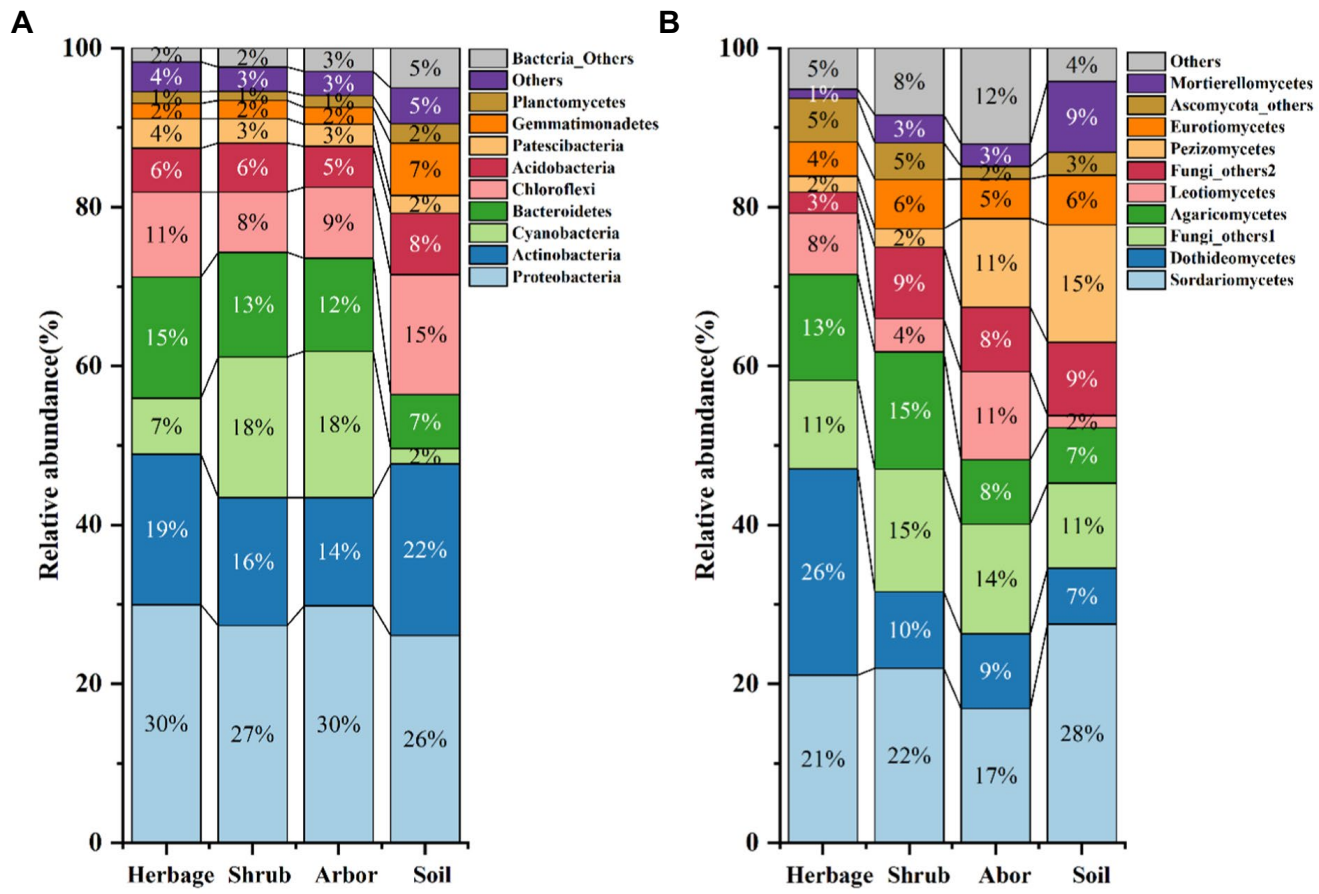
Relative abundance of the dominant bacteria phyla **(A)** and dominant fungus classes **(B)** across all soils. Soils are grouped by plant types. The “Bacteria_Others” refers to the sum of bacteria which has very low relative abundance, while “Others” refers to the unidentified sequences.

Using the high throughput sequencing platform, 21,884,403 quality sequences were obtained from the 54 soil samples of four types; among them, 86,366 were identified at 100% similarity, being mostly bacteria and 11,188 were identified at 100% similarity, being mostly fungi. At the bacterial phylum level, Proteobacteria (28.8%), Actinobacteria (16.7%), Cyanobacteria (13.1%), Bacteroidetes (12.7%), Chloroflexi (9.7%), and Acidobacteria (5.8%) were dominant, accounting for more than 80% of all sequences (Figure 1) Ascomycota was the most common fungal phylum among the samples (Figure 1). At the fungal class level, Sordariomycetes (21.9%), Dothideomycetes (13.0%), Agaricomycetes (10.8%), Pezizomycetes (7.5%), and Leotiomycete (6.1%) were dominant. Dothideomycetes abundance was the highest in the herb rhizosphere (Figure 1).

In the case of bacteria, compare to those in all the rhizosphere soils, most high abundance microbiomes were significantly enriched in bulk soil, whereas Cyanobacteria and Bacteroidetes abundances in bulk soil were significantly lower (Figure 1; Supplementary Table S4). The abundances of phylum Cyanobacteria in the herbs’ rhizosphere soil were also significantly lower than that in arbors’ rhizosphere soil, but herbs had the most Bacteroidetes than the others (Figure 1; Supplementary Table S4). And in the case of fungi, the abundance of classes Leotiomycetes in arbor rhizosphere was significantly higher than those in the other soils. The abundances of Sordariomycetes, Pezizomycetes and Mortierellomycetes in bulk soils were the highest while the abundances of class Dothideomycetes and Leotiomycetes in bulk soils were the lowest among the different groups (Figure 1; Supplementary Table S5).

### 3.2. Effect of vegetation type on soil microbial community structure

Although there were no obvious differences in fungal α diversity among the groups, there were significant differences in bacterial α diversity. The bacterial α diversity of herbs and bulk soils were significantly higher than those of shrubs and arbors. And there is no obvious difference in bacterial diversity between herbs and bulk soils, nor between shrubs and arbors (Supplementary Figures S2, S3).

**Figure 2:**
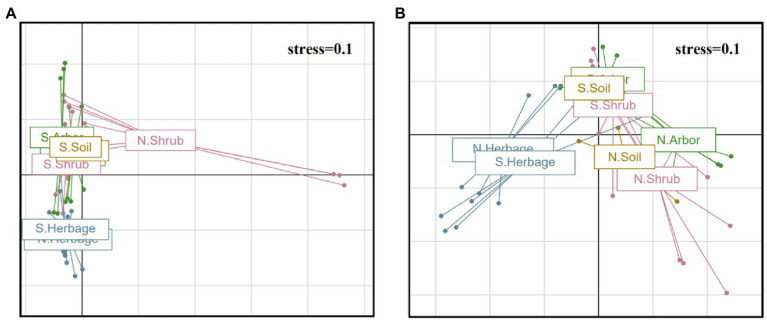
Non-metric multidimensional scaling (NMDS) of the bacterial **(A)** and fungal **(B)** community among the samples.

According to the results of VPA, the explanation of plant type (1.1% for bacteria and 3.4% for fungi) for the differences between groups was higher than that of location (0.5% for bacteria and 1.7% for fungi; Supplementary Table S7). In addition, both the NMDS and Multivariate Welch ANOVA showed obvious differences between bacteria and fungus (Figure 2; Supplementary Tables S8, S9). According to the results of Multivariate Welch ANOVA, except for some comparisons (S. Herbage vs. N. Herbage, N. Bulk soil vs. N. Shrub, S. Bulk soil vs. N. Shrub, S. Bulk soil vs. N. Arbor, S. Bulk soil vs. N. Bulk soil, S. Bulk soil vs. S. Arbor and S. Arbor vs. S. Shrub), the bacteria in most comparisons had significant differences between groups (Figure 2A; Supplementary Table S8). While the majority of the differences of fungi among groups were significant, except for the differences between the bulk soil, the rhizosphere soil of shrubs and arbors in the south and between the bulk soil in the south and in the north (Figure 2B; Supplementary Table S9). Furthermore, Mantel test results showed that plant phylogeny had a strong influence on bacterial community structure, and not on fungal community structure, while plant vegetation types have a significant impact on the difference between groups of both bacteria and fungi (Supplementary Table S6).

**Figure 3:**
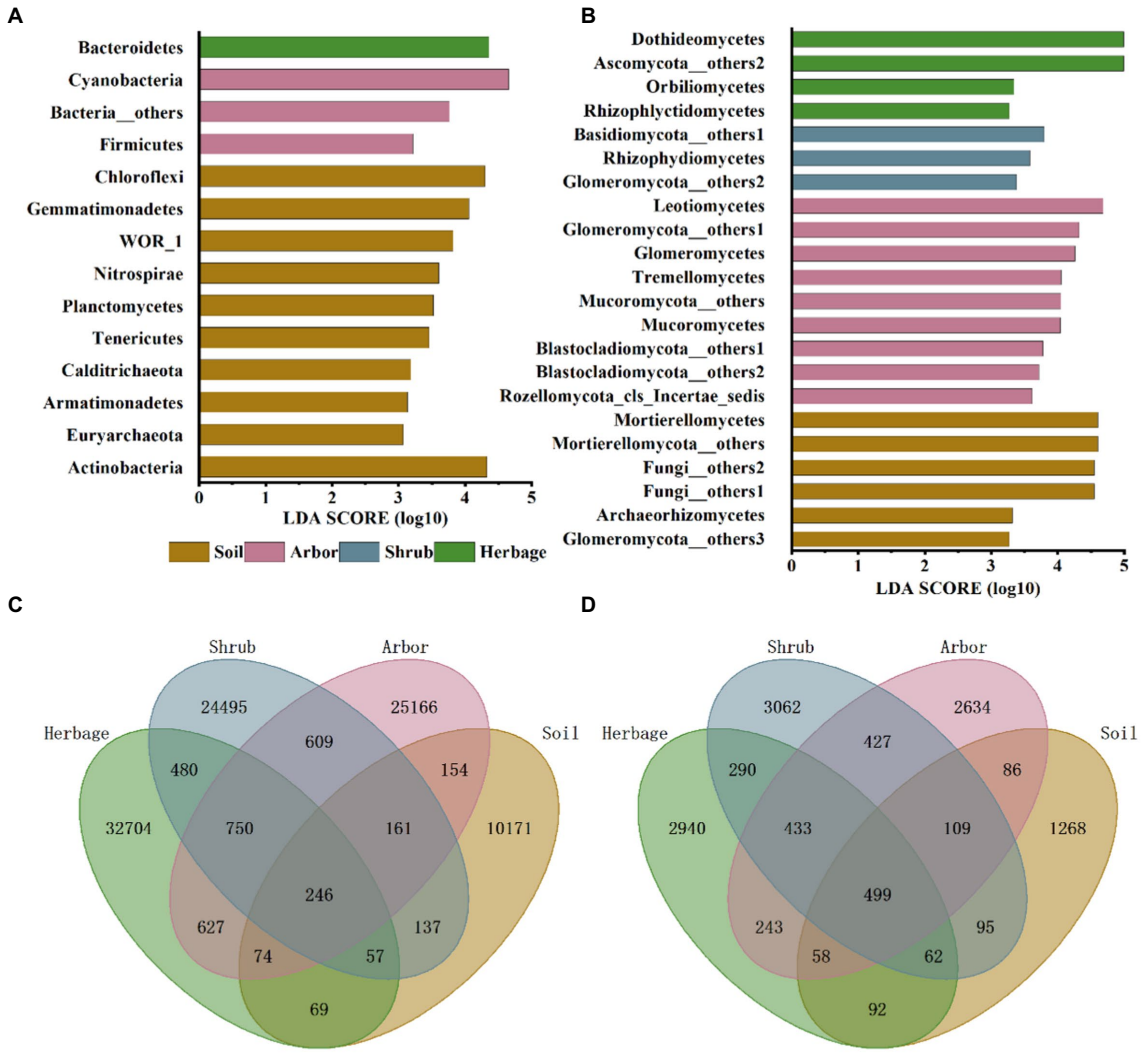
Microbial community composition in different soil samples. Linear discriminant analysis effect size (LEfSe) of bacteria phyla/ phylum **(A)** and fungus phyla/ classes **(B)** of four plant types. Venn diagrams of bacteria **(C)** and fungi **(D)** of four plant types.

Among bacteria, Cyanobacteria and Firmicutes were significantly higher in the rhizosphere soil of arbor. Bacteroidetes were significantly higher in herbaceous plants, while bulk soil had the largest number of endemic bacterial phyla (Figure 3A). In particular, the bulk soil contains a variety of archaea that are less abundant in the rhizosphere of plants.

For fungus (Figure 3B), Archaeorhizomycetes and Mortierellomycetes were significantly higher in soil than in rhizosphere soil of all plants. Leotiomycetes, Glomeromycota, Tremellomycetes, Rozellomycota and Mucoromycota were significantly higher in arbor soil. The Rhizophydiomycetes, Basidiomycota and Glomeromycota in the rhizosphere soil of shrub were significantly higher. Rhizophlyctidomycetes, Ascomycota and Orbiliomycetes were significantly higher in the rhizosphere soil of herbage.

According to the Venn plot (Figures 3C, D), although the number of bacteria involved in the analysis was much higher than the number of fungi, the number of common fungi in the four samples was still much higher than the number of common bacteria. In addition, the shrub rhizosphere soil had a higher number of common bacteria with arbor rhizosphere soil than with herbage rhizosphere soil, with an opposite trend observed in the case of fungi. In general, the shrub rhizosphere soil had the fewest specialized microbes.

### 3.3. Soil bacterial and fungal assembly in the four plant types

**Figure 4:**
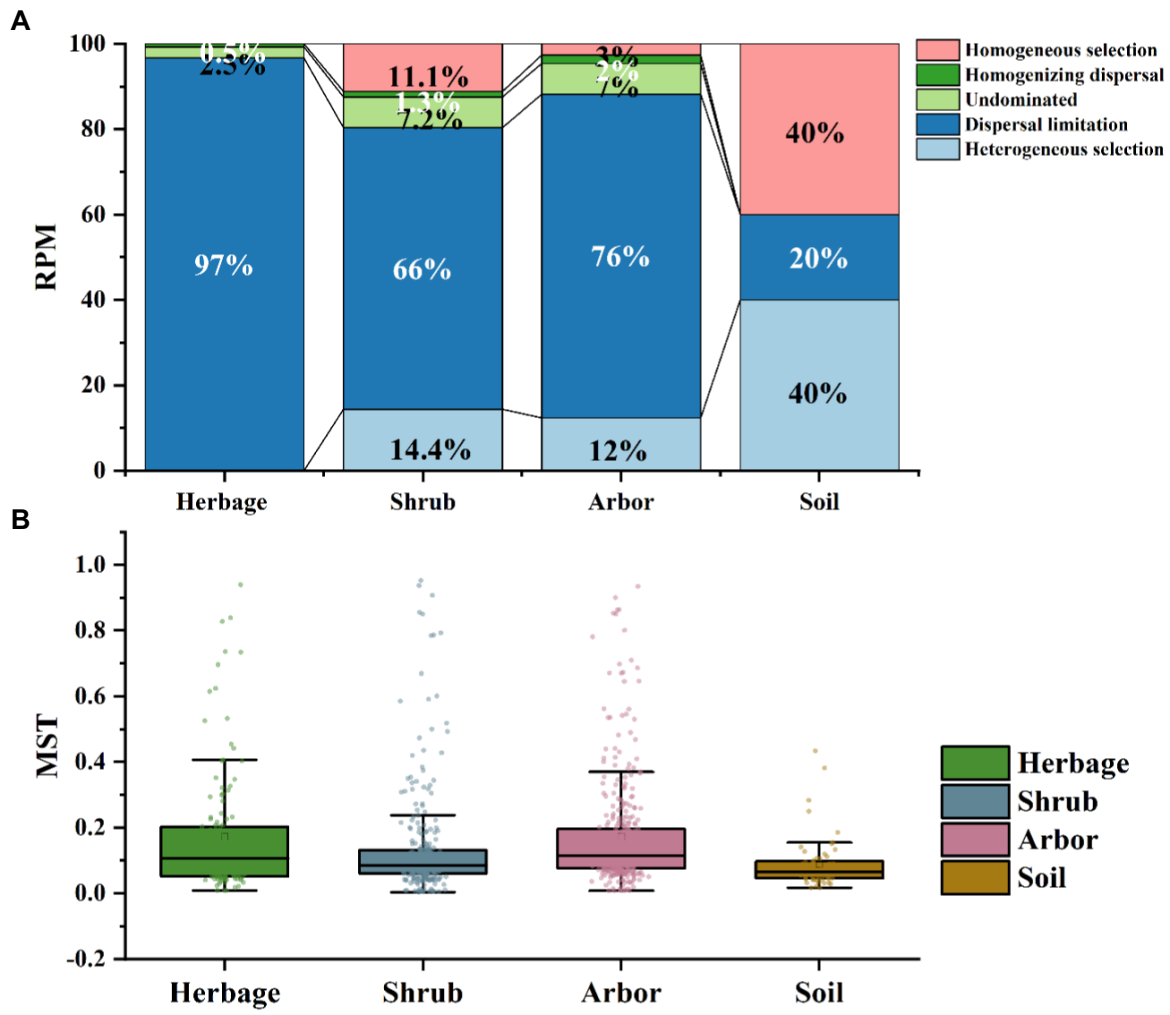
Assembly processes of soil microbiome in the four plant types. **(A)** Assembly processes of soil bacteria based on the combined method of ß-NTI. **(B)** Normalized stochasticity ratio (NST) of soil fungal in the four types of samples.

Bacterial community assembly in rhizosphere was dominated by stochasticity processes, and dispersal limitation was more prevalent in the rhizosphere than in the bulk soil, especially in the herb rhizosphere soil (Figure 4A). Both arbor and shrub rhizosphere soils were partially dominated by stochasticity processes. And in bulk soil, bacterial community assembly was more dominated by deterministic process (Figure 4B). In addition, deterministic processes dominated fungal community assembly processes across all samples. Among them, the assembly processes of shrub rhizosphere were the closest to those of the bulk soil, with relatively similar Normalized Stochasticity Ratio values (Figure 4B).

### 3.4. Effect of vegetation type on molecular ecological networks of microbial communities

**Figure 5:**
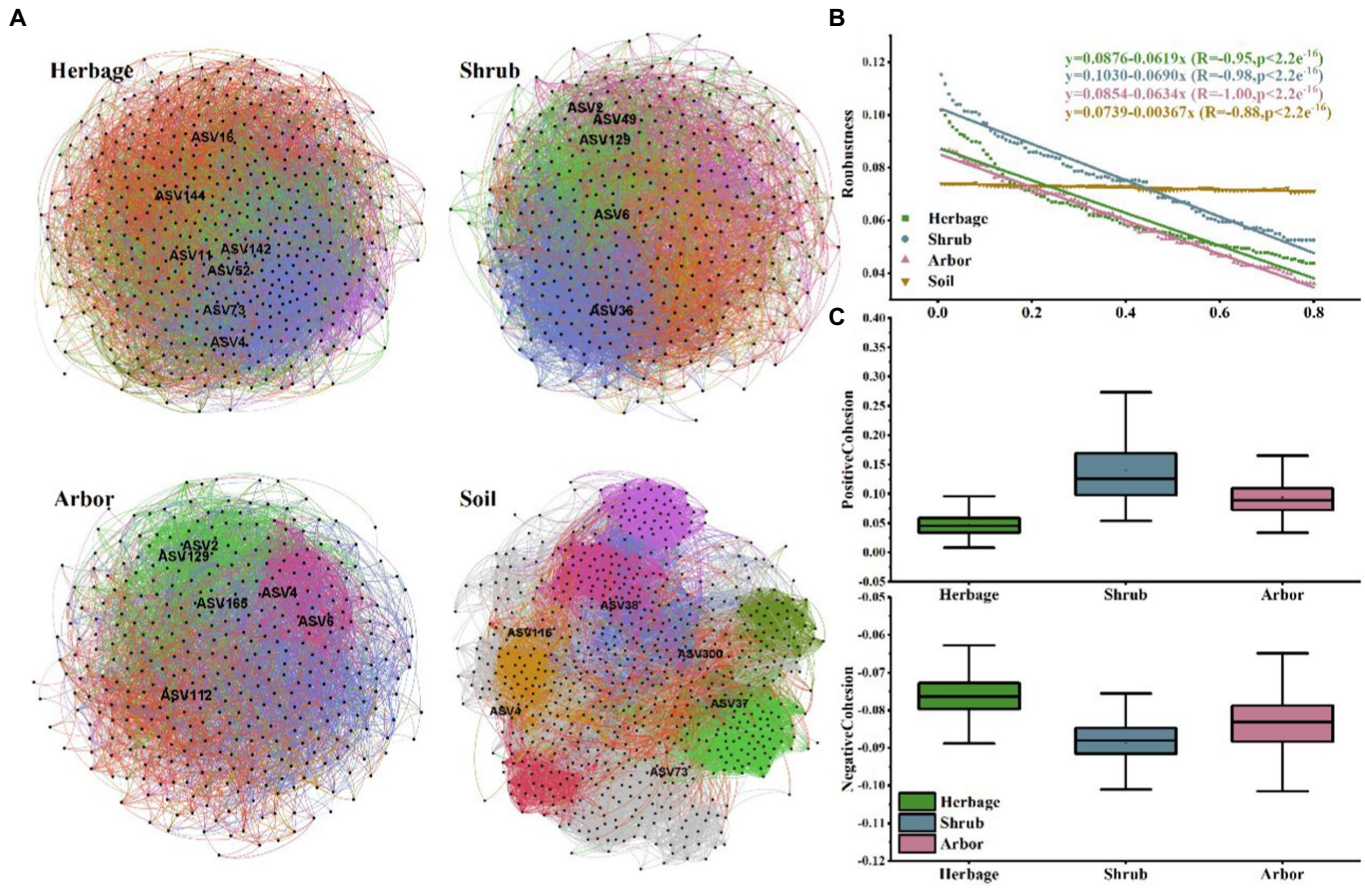
**(A)** Plant rhizosphere networks in the four sample types. Networks represent random matrix theory co-occurrence models, where nodes represent ASVs, and the edges between the nodes indicate significant correlations. In each panel, the size of each node is proportional to the number of connections (i.e., node degree) and the edge color indicates that the node belongs to a different module. **(B)** Robustness of microbial community in the rhizosphere of three types of plants. **(C)** Cohesion of microbial community in the rhizosphere of three types of plants.

Individual networks were constructed for each of the four sample types (Figure 5A; Table 1). Compared with rhizosphere soil, bulk soil had a more complex microbial network, with the most positive and negative connections. The rhizosphere soils of different vegetation types had different core flora. There were 7, 5, 6 and 6 keystone species in the herb, shrub, and arbor rhizosphere soils, and bulk soils, respectively (Table 2). Among them, shrub had the fewest core bacteria. The node and edge numbers in the networks decreased in the order of bulk soil, and herbage, shrub, and arbor rhizosphere soils. Similar trends were observed in the number of positive correlations and negative correlations, and average connectivity (avgK). However, with regard to the ratio of positive to negative correlations and average clustering coefficient (avgCC), shrub rhizosphere had the largest values, excluding bulk soil. In addition, shrub rhizosphere has the lowest average path distance (GD), the lowest number of modules, and the highest Graph density. In addition, the network modularity increased in the order of herbs, shrub, and arbor rhizosphere soils, and bulk soil.

**Table 1:** Topological properties of the empirical molecular ecological networks of microbial communities in groups.

Network metrics	Type			
	Herbage	Shrub	Arbor	Soil
Number of nodes	533	401	393	754
Number of edges	10,088	6,815	5,233	16,743
Number of positive correlations	5,350	3,738	2,764	11,213
Number of negative correlations	4,738	3,077	2,469	5,530
Ratio of positive to negative correlations	1.129	1.215	1.119	2.028
Average connectivity (avgK)	18.927	16.995	13.316	22.206
Average path distance (GD)	2.224	2.206	2.309	2.640
Average clustering coefficient (avgCC)	0.278	0.311	0.262	0.546
Graph density	0.071	0.085	0.068	0.059
Number of modules^a^	9	9	12	17
Modularity	0.285	0.318	0.352	0.648

a Number of modules with ≥ 5 nodes in the networks.

**Table 2:** Microbial community composition of the keystone species.

Group	ASVID	Category	Kingdom	Phylum	Class
Herbage	ASV4	Provincial hubs	k__Plantae	p__unidentified	c__unidentified
	ASV11	Provincial hubs	k__Fungi	p__Ascomycota	c__Sordariomycetes
	ASV16	Provincial hubs	k__Fungi	p__Ascomycota	c__Sordariomycetes
	ASV52	Provincial hubs	k__Fungi	p__Ascomycota	c__Sordariomycetes
	ASV73	Provincial hubs	k__Fungi	p__Ascomycota	c__Dothideomycetes
	ASV142	Provincial hubs	k__Fungi	p__Ascomycota	c__Dothideomycetes
	ASV144	Provincial hubs	k__Fungi	p__Ascomycota	c__Dothideomycetes
Shrub	ASV2	Provincial hubs	Unassigned		
	ASV6	Provincial hubs	k__Fungi	p__Ascomycota	c__Sordariomycetes
	ASV36	Provincial hubs	k__Plantae	p__unidentified	c__unidentified
	ASV49	Provincial hubs	k__Fungi	p__unidentified	c__unidentified
	ASV129	Provincial hubs	k__Fungi		
Arbor	ASV2	Provincial hubs	Unassigned		
	ASV4	Provincial hubs	k__Plantae	p__unidentified	c__unidentified
	ASV6	Provincial hubs	k__Fungi	p__Ascomycota	c__Sordariomycetes
	ASV112	Provincial hubs	k__Fungi	p__Ascomycota	c__Leotiomycetes
	ASV129	Provincial hubs	k__Fungi		
	ASV165	Provincial hubs	k__Fungi	p__unidentified	c__unidentified
Soil	ASV4	Provincial hubs	k__Plantae	p__unidentified	c__unidentified
	ASV37	Provincial hubs	k__Fungi	p__Ascomycota	c__Pezizomycetes
	ASV38	Provincial hubs	k__Fungi	p__Basidiomycota	c__Tremellomycetes
	ASV73	Provincial hubs	k__Fungi	p__Ascomycota	c__Dothideomycetes
	ASV116	Provincial hubs	k__Plantae		
	ASV300	Provincial hubs	k__Fungi	p__Ascomycota	c__Sordariomycetes

Network robustness was also examined. The results indicated a more stable microbial network in the bulk soil than in the rhizosphere soils. Nodes and edges were discarded in declining order of node betweenness. Therefore, we observed that the natural connectivity of networks in all vegetation types exhibited sharp slopes in all the samples excluding in the bulk soil, suggesting poor stability (Figure 5B). Furthermore, for each group, z-scores and c-scores were calculated for the nodes in the network to identify the keystone species.

Additionally, the shrub has the highest positive cohesion and the lowest negative cohesion (Figure 5C), indicating more cooperation and less competition between microbes in the shrub rhizosphere than in those of other vegetation types.

## 4. Discussion

Considering the significant effects of human activities on soil environments and vegetation diversity, it is necessary to explore the influence of vegetation type on microbial communities, and whether bacteria and fungi respond differently, at local environment scales. Such a study could provide a scientific basis for understanding microbial function over small spatial scales. In the present study, the influence of vegetation type on soil microbial community structure was glaringly obvious, especially in the case of rhizosphere microbes. Our results showed that rhizosphere microbial community structure could differ considerably across different vegetation types (arbor, shrub, and herbs), which are frequently disturbed by human activities.

### 4.1. Rhizosphere bacterial and fungal community were significantly different across vegetation types

Arbor, shrub, and herbs are the most common vegetation assemblages in city parks or campuses; therefore, city parks and campuses are ideal sites for investigating the impact of aboveground vegetation type on belowground soil microbial community structure, while eliminating the effect of climate and soil type. Arbors refer to trees with an upright trunk, usually 6–10 m high, with a trunk independent from the root, and a clear distinction between the trunk and the crown. Arbors also have strong vitality and are widely distributed. At present, arbors are basically found in all terrestrial biomes, including desert, Arctic, and other harsh environments (McBride and Douhovnikoff, 2012; Zhang et al., 2022). Conversely, shrubs are short plants (usually <6 m) with no obvious trunk and numerous branches near the ground, most of which are clustered. Shrubs are generally broad-leaved plants, and some coniferous plants, such as juniper, are shrubs (Lenard, 2008). Shrubs are widely distributed globally, mostly in the tropics and subtropics, and can also be found in arid regions (Xu et al., 2020). In China, shrubs are mainly spread in Zhejiang, Jiangsu, Anhui, Henan, etc., covering about 20% of the land area (Piao et al., 2009). Although there are many differences between arbors and shrubs, they have many similarities with regard to growth habit (Jingui et al., 2023). They are both perennial plants and can survive more than 3 years. Moreover, arbors and shrubs have large numbers of lignified cells, which are obviously different from herbs (Crivellaro et al., 2022). Herbs are usually short with stems that are soft and that break easily. In addition, many herbs are annuals, biennials or triennials, and their xylems are not developed and the vascular bundles do not have cambiums, so that they cannot grow continuously (Evans and Ortega, 2019). Herbs are generally adapted to warm and humid environments. However, herbaceous plants are very resilient, and can be found in hot and humid areas, as well as cold and dry areas. On the whole, the most obvious differences among trees, shrubs, and herbs are based on their physiological traits, biomass, and life span (Yuan et al., 2020).

In the present study, *Pinus massoniana, Gingko biloba, Solanum nigrum, Ligustrum lucidum, Forsythia viridissima, Veronica persica, Punica granatum, Cercis chinensis, Bischofia polycarpa, Oxalis corniculata, Eriobotrya japonica, Euonymus japonicus, Lonicera maackii* and *Ophiopogon japonicus* are grouped into arbors, shrubs, and herbs.

Due to differences in individual size, life history, or physiological function, bacteria and fungus exhibit distinct responses to aboveground plants. In the present study, we observed that soil bacterial α diversity was more sensitive to vegetation type than fungal α diversity. Bacteria are minute, propagate rapidly, and form spores, so that they are ubiquitous and easily dispersed (Foissner, 2006). Conversely, fungi proliferate mainly through budding and spore reproduction. Fungus can also form mycelia, with diverse functions (Cairney and Burke, 1996; Hodge, 2000). Fungal communities jointly form complex belowground networks, which drive the establishment of plant populations and communities, as well as soil nutrient turnover (Yang et al., 2022). According to Sheldrake (2020), fungi are regenerators, recyclers, and network builders that connect the world. Therefore, one or similar fungal species can be observed in different vegetation rhizosphere soils, so that we did not investigate the significant effect of vegetation type on fungal α diversity.

In the β diversity analysis, the results of VPA showed that the explanation of vegetation type for the difference is greater than that of location, which is partially different from the findings of Vieira et al. (2020). In addition, Yang et al. (2019) found that the influence of plant factors on rhizosphere microbial community was greater than spatial factors in the forests of eastern China. In our study, the campus garden is subject to periodic management and frequent human activities, and this will reduce the difference of soil between the two gardens. On the other hand, with the strong effect of host plants, plants exhibited stronger effect than the locations. Additionally, given the low percentage of the variance between samples that explain both the type of plant and the sampling location, possibly, other unaccounted factors can help explain the differences between the samples. According to the results of Multivariate Welch ANOVA, we found that the differences between rhizosphere soils of different vegetation types were generally significant. Consistent with Fitzpatrick et al.’s (2018) study, which found the plant species showed strong effect on the rhizospheric communities. This is also partly similar to the findings in the study of the low Arctic tundra (Shi et al., 2015) which found the soil microbial community could be differed by vegetation types. This may be due to the fact that shrubs and herbs are often closely interlaced, and these interactions would reduce the difference between groups.

### 4.2. Contrasting bacterial and fungal community assembly processes between bulk and rhizosphere soil

In the present study, bulk soil and rhizosphere soil bacterial communities showed contrasting assembly processes, even under different vegetation types. Bacterial community assembly in bulk soil was more dominated by deterministic process, whereas the rhizosphere bacterial community assembly was dominated by stochasticity and the construction of fungal communities was all dominated by deterministic processes (Figure 4). Similar phenomena were observed in a wheat field ecosystem, with deterministic factors playing a greater role in the assembly of nitrogen fixing bacteria communities in the bulk soil than in the rhizosphere soil (Fan et al., 2018). While Yang et al. (2018) reported a contrary phenomenon that deterministic processes played a more important role than stochastic processes in bacterial community assembly processes in Chinese grassland ecosystem. This may be because environmental filtration has a greater impact on the biogeographic pattern of bacteria. The Anna Karenina principle could explain why we observed that rhizosphere bacterial community assembly was dominated by stochastic process. According to the principle, healthy hosts have relatively stable microbial communities, which form close clusters in an orderly space, while various external stress factors undermine such stability, leading to more dispersed microbial communities (Zaneveld et al., 2017). Therefore, the rhizosphere filtering effect would lead to the cultivation of specific species and be accompanied by the Anna Karenina principal effect, so that a random process occurs.

### 4.3. Rhizosphere soil harbor less complex networks than bulk soil

Due to the strong filtering effect of plant roots, they harbor simpler communities than bulk soil, and in turn, less complex association networks. Consistent with our study, in farmland, Fan et al. (2018) observed that the network structure of nitrogen fixing microbial communities in rhizosphere soil was less competitive and more stable than that in bulk soil. In grasses, researchers found that rhizosphere networks had less nodes and edges, lower density, but had higher modularity, and greater positive links than bulk soil networks (Li et al., 2021). In a forest ecosystem, co-occurrence network analysis detected relatively higher network complexity and node connectivity in bulk soil than in the rhizosphere community (Jing et al., 2022; Wang et al., 2022). These studies indicated that less complex association networks were prevalent in rhizosphere. In addition, Ling et al. (2022) analyzed 557 pairs of published 16S rDNA amplification sequences from non-rhizosphere soil and rhizosphere soil of different ecosystems globally, and found that the rhizosphere had relatively reduced microbial diversity due to the selection of corresponding microbial populations from soil seed banks, thus forming a highly modular but unstable bacterial network in the rhizosphere. This indicate that less complex networks are not related to the community stability, it might be depending on the ecosystem type.

### 4.4. Rhizosphere bacterial community structure significantly correlated with plant phylogeny

Yang et al. (2019) observed soil fungal communities could be strongly influenced by plant phylogenetic distance in forest ecosystems across Eastern China. In the present study, we also observed that rhizosphere bacterial communities were significantly correlated with plant phylogeny. In addition, in root microbiomes of multiple plant phyla (Yeoh et al., 2017), researchers observed that soil bacterial communities could be strongly affected by plant phylogeny at the small scale. The reason could be that, at very small scales, plants exert very strong effects on bacteria due to their lower interrelationships, and fungi, because of their hyphae, build highly connected networks at the local scale, so that plant phylogeny did not exhibit strong effects with regard to fungal community structure. Beside this, the effect of specific sampling quantity (Hermans et al., 2019) and soil depth (Chu et al., 2016) on the rhizosphere community were also reported, these two potential impacting factors will be tested in the future.

## 5. Conclusion

Overall, our results showed that rhizosphere bacterial and fungal community structure could vary across vegetation types in a small scale, and that bacterial assembly was dominated by stochasticity while deterministic processes dominated fungal community assembly processes. Rhizosphere associated networks showed less complexity than bulk soil networks, and their keystone species varied across vegetation types. Community dissimilarities of total bacteria could be influenced by plant phylogenetic distance, while fungi showed no significant correlation. The results of the present study provide insights on belowground microbial structure at the local environment scale under different vegetation types, and might facilitate the knowledge of conservation of belowground microbial biodiversity at a local environment scale.

## Data availability statement

The original contributions presented in the study are publicly available. This data can be found at: NCBI, PRJNA898095.

## Author contributions

YS and LL designed the study. YS, XL, LM, MZ, BL, and LL performed the research and analyzed the data. LL, LM, MZ, and YS wrote, edited, and finalized the manuscript. All authors contributed to the article and approved the submitted version.

## Funding

This work was supported by grants from the Natural Science Foundation of Henan (grant numbers 222300420035, 212300410112) and the National Natural Science Foundation of China (grant number 42077053).

## Conflict of interest

The authors declare that the research was conducted in the absence of any commercial or financial relationships that could be construed as a potential conflict of interest.

## Publisher’s note

All claims expressed in this article are solely those of the authors and do not necessarily represent those of their affiliated organizations, or those of the publisher, the editors and the reviewers. Any product that may be evaluated in this article, or claim that may be made by its manufacturer, is not guaranteed or endorsed by the publisher.

## Supplementary material

The Supplementary material for this article can be found online at: https://www.frontiersin.org/articles/10.3389/fmicb.2023.1129471/full#supplementary-material

## Footnotes

^1^ http://www.magigene.com/

^2^ https://unite.ut.ee/
