# Supplementary material for: Rhizosphere microbial community assembly and association networks strongly differ based on vegetation type at a local environment scale
Source: Front Microbiol. 2023 Mar 14;14:1129471. doi: 10.3389/fmicb.2023.1129471 (PMC10043216; doi:10.3389/fmicb.2023.1129471)

Table S1. Sequences of the primers for bacteria, fungi, and arbuscular mycorrhizal fungi.

	N	S
**1**	Ligustrum lucidum	Ligustrum lucidum
**2**	Forsythia viridissima	Forsythia viridissima
**3**	Oxalis corniculate	Oxalis corniculate
**4**	Veronica persica	Veronica persica
**5**	Bischofia polycarpa	Eriobotrya japonica
**6**	Ginkgo biloba	Pinus massoniana
**7**	Cercis chinensis	Lonicera maackii
**8**	Punica granatum L	Euonymus japonicus
**9**	Solanum nigrum	Ophiogon japonicus.

Table S2. Sequences of the primers for bacteria, fungi, and arbuscular mycorrhizal fungi.

Soil microbes	Primer code	Primer sequence
**Bacteria**	**338F**	5’-ACTCCTACGGGAGGCAGCA-3’
	**806R**	5’-GGACTACHVGGGTWTCTAAT-3’
**Fungi**	**ITS3F**	5’-GCATCGATGAAGAACGCAGC-3’
	**ITS4R**	5’-TCCTCCGCTTATTGATATGC-3’

Table S3. GenBank number for plastome sequences used in the present study.

Species	GenBank number
Oxalis corniculata	NC051971
Bischofia polycarpa	MZ826267
Euonymus japonicus	MZ567073
Cercis chinensis	MZ128523
Eriobotrya japonica	NC034639
Punica granatum	MT600023
Forsythia viridissima	MW856917
Ligustrum lucidum	MH394207
Veronica persica	NC031344
Solanum nigrum	KM489055
Lonicera maackii	MN256451
Ophiopogon japonicus	MK952744
Gingko biloba	MN443423
Pinus massoniana	MF564195

Table S4. Significance of the relative abundance of dominant bacteria phyla in all soils. Values in the same row with different alphabetic superscripts (a, b, c) are significantly different between the four groups.

	**Type**			
	**Herbage**	**Shrub**	**Arbor**	**Soil**
**Proteobacteria**	0.30^{a}	0.27^{a}	0.30^{a}	0.26^{a}
**Actinobacteria**	0.19^{a}	0.16^{ab}	0.14^{b}	0.22^{a}
**Cyanobacteria**	0.07^{b}	0.18^{ab}	0.18^{a}	0.02^{c}
**Bacteroidetes**	0.15^{a}	0.13^{ab}	0.12^{b}	0.07^{c}
**Chloroflexi**	0.11^{b}	0.08^{c}	0.09^{bc}	0.15^{a}
**Acidobacteria**	0.06^{b}	0.06^{ab}	0.05^{b}	0.08^{a}
**Patescibacteria**	0.04^{a}	0.03^{a}	0.03^{a}	0.02^{a}
**Gemmatimonadetes**	0.02^{b}	0.02^{b}	0.02^{b}	0.07^{a}
**Planctomycetes**	0.01^{b}	0.01^{b}	0.01^{b}	0.02^{a}
**Others**	0.04^{a}	0.03^{b}	0.03^{b}	0.05^{a}
**Bacteria_ Others**	0.02^{c}	0.02^{bc}	0.03^{b}	0.05^{a}

Table S5. Relative abundances of dominant fungal class in all soils. Values in the same row with different alphabetic superscripts (a, b, c) are significantly different between the four groups.

	Type			
	**Herbage**	**Shrub**	**Arbor**	**Soil**
**Sordariomycetes**	0.21^{ab}	0.22^{ab}	0.17^{b}	0.28^{a}
**Dothideomycetes**	0.26^{a}	0.1^{b}	0.09^{b}	0.07^{b}
**Fungi_others1**	0.11^{b}	0.15^{a}	0.14^{ab}	0.11^{ab}
**Agaricomycetes**	0.13^{a}	0.15^{a}	0.08^{a}	0.07^{a}
**Leotiomycetes**	0.08^{a}	0.04^{ab}	0.11^{a}	0.02^{b}
**Fungi_others2**	0.03^{b}	0.09^{a}	0.08^{a}	0.09^{a}
**Pezizomycetes**	0.02^{b}	0.02^{ab}	0.11^{a}	0.15^{a}
**Eurotiomycetes**	0.04^{a}	0.06^{a}	0.05^{a}	0.06^{a}
**Ascomycota_others**	0.05^{a}	0.05^{ab}	0.02^{b}	0.03^{ab}
**Mortierellomycetes**	0.01^{c}	0.03^{ab}	0.03^{b}	0.09^{a}
**Others**	0.05^{b}	0.08^{ab}	0.12^{a}	0.04^{b}

Table S6. Correlation between genetic distance or plant vegetation type and bacterial or fungal community structure determined based on the Mantel test (P <0.05, permutation = 999). Significant values are in bold.

	Bacteria		Fungi	
	**r**	**p**	**r**	**p**
**Genetic distance**	0.317	**0.001**	0.116	0.052
**Plant vegetation type**	0.091	**0.002**	0.151	**0.001**

Table S7. The Variance Partitioning Analysis (VPA) of the bacterial and fungal community among the samples.

	Location	Plant Type
Bacteria	0.005	0.011
Fungi	0.017	0.034

Table S8. Multivariate Welch ANOVA (P <0.05, permutation = 99999) among bacteria between all groups.

	N.Herbage	N.Shrub	N.Arbor	N. Bulk soil	S.Herbage	S.Shrub	S.Arbor
**N.Herbage**							
**N.Shrub**	**0.001**						
**N.Arbor**	**0.001**	**0.001**					
**N.Bulk soil**	**0.007**	0.062	**0.036**				
**S.Herbage**	0.112	**0.001**	**0.001**	**0.013**			
**S.Shrub**	**0.001**	**0.003**	**0.001**	**0.006**	**0.003**		
**S.Arbor**	**0.001**	**0.001**	**0.007**	**0.010**	**0.001**	0.071	
**S.Bulk soil**	**0.004**	0.160	0.113	0.088	**0.010**	**0.027**	0.067

Table S9. Multivariate Welch ANOVA (P <0.05, permutation = 99999) among fungi between all groups.

	**N.Herbage**	**N.Shrub**	**N.Arbor**	**N. Bulk soil**	**S.Herbage**	**S.Shrub**	**S.Arbor**
**N.Herbage**							
**N.Shrub**	**0.001**						
**N.Arbor**	**0.001**	**0.001**					
**N. Bulk soil**	**0.018**	**0.035**	**0.030**				
**S.Herbage**	**0.037**	**0.001**	**0.001**	**0.021**			
**S.Shrub**	**0.001**	**0.004**	**0.001**	**0.011**	**0.014**		
**S.Arbor**	**0.001**	**0.001**	**0.001**	**0.017**	**0.006**	0.254	
**S. Bulk soil**	**0.022**	**0.031**	**0.030**	0.109	**0.030**	0.063	0.125

FIGURE S1. The schematic diagram of sample collection location(a) (Sampling map was download from https://www.henu.edu.cn/zjhd/xqsyt.htm and was modified accordingly) and the phylogenetic tree of 14 species collected in the experiment(b).

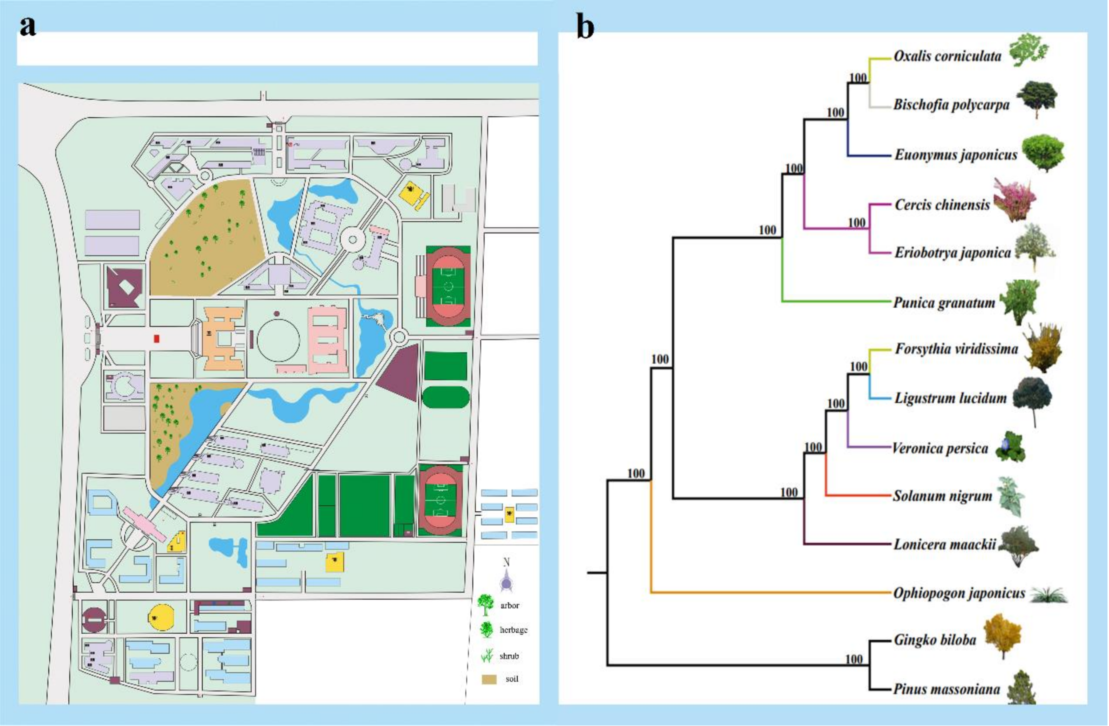

FIGURE S2. α-Diversities of soil bacteria in the four plant types. Shannon(a), Simpson(b), Observed(c) and Chao 1(d) indices of the fungus of rhizosphere soil.

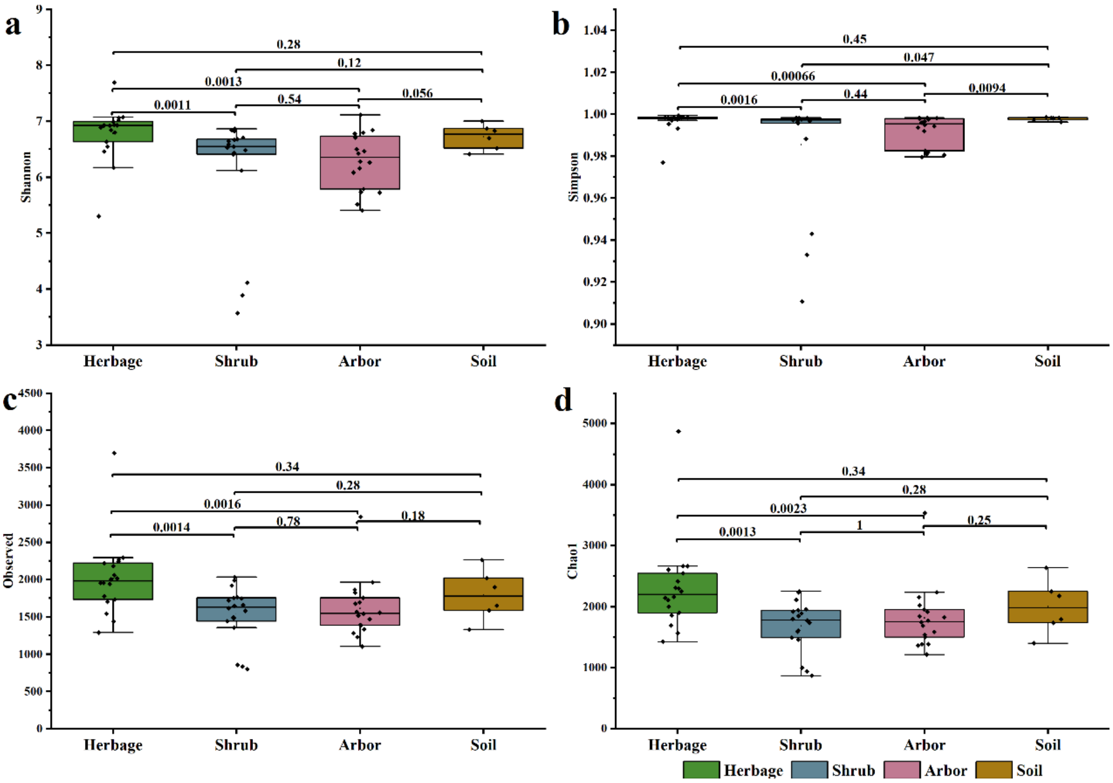

FIGURE S3. α-Diversities of soil fungus in the four plant types. Shannon(a), Simpson(b), Observed(c) and Chao 1(d) indices of the bacteria of rhizosphere soil.

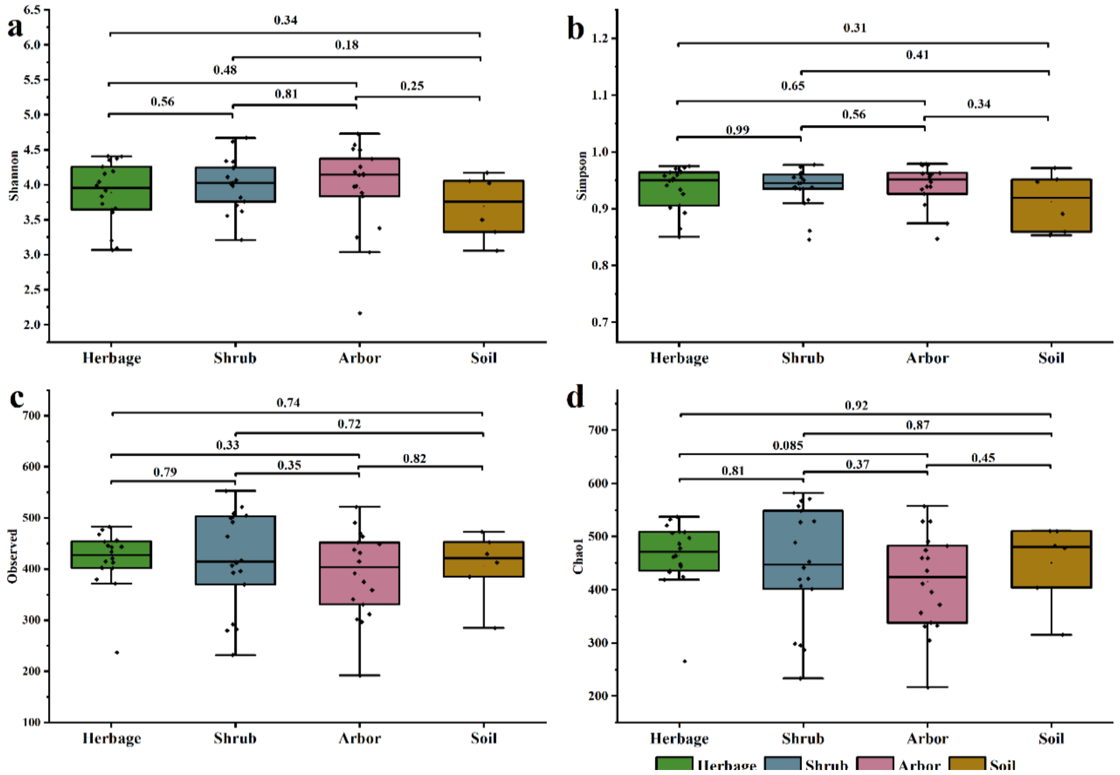

FIGURE S4. Principal coordinate analysis (PCoA) of the bacterial (a) and fungal (b) community among the samples based on Bray-Curtis distances.

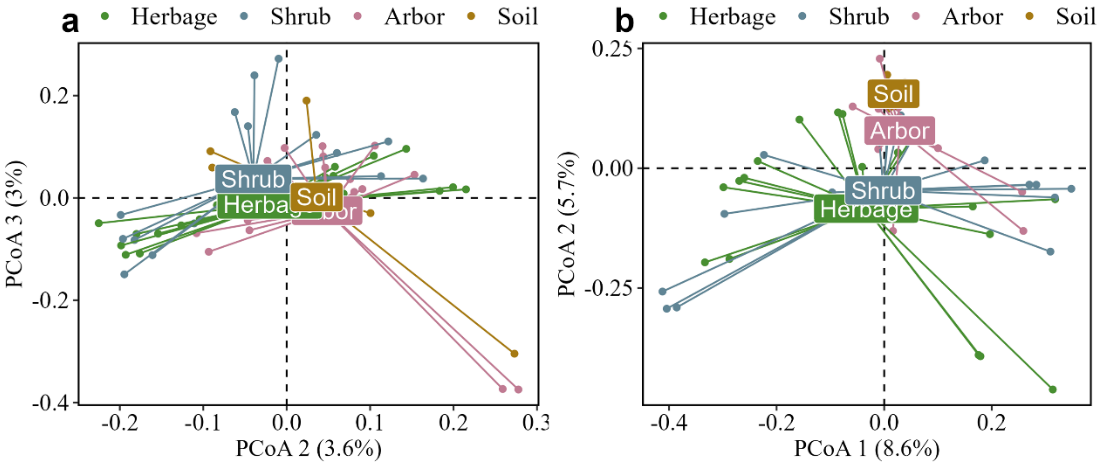

FIGURE S5. Principal component analysis (PCA) of the bacteria (a) fungus (b) among the samples. There was no significant grouping of fungus and bacteria in the four plant types.

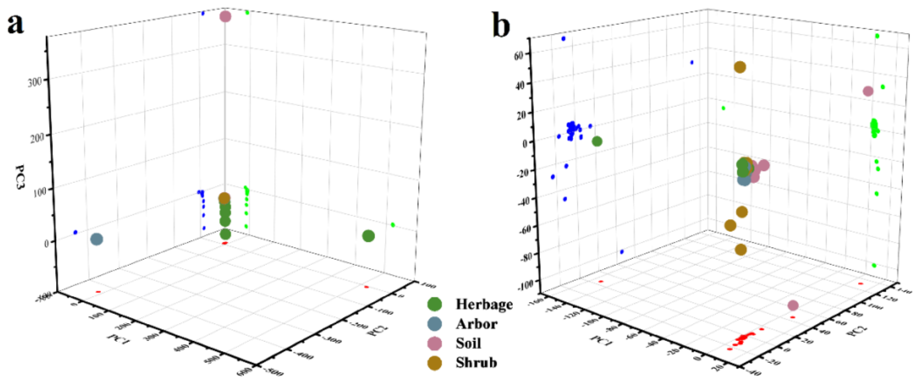

FIGURE S6. Principal coordinate analysis (PCoA) of the bacteria (a) fungus (b) between samples from two sampling locations.

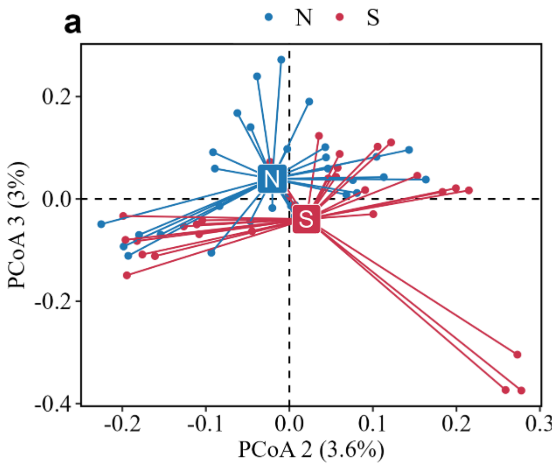

Supplement: Supplementary file 1 [file Data_Sheet_1.pdf]
